# Collaborating with the people who experience psychosis: From subjects to partners

**DOI:** 10.1192/j.eurpsy.2023.1314

**Published:** 2023-07-19

**Authors:** N. D. Semenova

**Affiliations:** Moscow Research Institute of Psychiatry, Moscow, Russian Federation

## Abstract

**Introduction:**

Despite the critical role of motivation in psychosocial treatment and rehabilitation, as well as functional outcomes in schizophrenia, service user voices are not always present in setting research agendas on this topic. This is important since the service user’s involvement in the research process helps prioritize research questions (Wykes et al., 2015).

**Objectives:**

We have begun a consultation process to bridge the gap between research and practice on ‘motivation in schizophrenia.’ The study’s main objective was to produce the means to increase motivation in schizophrenia from the perspective of users.

**Methods:**

In the current study, we asked the service users about the priorities for ‘motivation and schizophrenia’ research and also suggested involving service users in research itself as partners. Expert Panels with peer leaders previously involved in psychosocial rehabilitation programs took place. A total of 12 Panels (group meetings) were held during the year with three users (schizophrenia spectrum outpatients) aged 45, 47, and 50, male. The reports were analysed using content analysis to generate main themes and findings (Braun & Clarke, 2006).

**Results:**

The users’ elements of motivation may differ from organizationally defined ones; this may be related to different opinions and ethical standards among clinicians on patients’ autonomy and right to refuse treatment. A discussion of the topics patients have developed to improve the motivation, engagement, and management of patients with schizophrenia in psychosocial treatment and rehabilitation is presented.

**Conclusions:**

The approach successfully generated items for questionnaires that usesr participants declared with a sence of pride in and ownership of. It is, therefore, possible to create measures of motivation that users feel reflect their understanding and experiences. The outcome of this research is that other Russian researchers will be inspired to follow the same patrnership path and encourage service users to participate rather than be subjected to research.

**Disclosure of Interest:**

None Declared

